# Haemosporida prevalence and diversity are similar in endangered wild whooping cranes (*Grus americana*) and sympatric sandhill cranes (*Grus canadensis*)

**DOI:** 10.1017/S0031182016002298

**Published:** 2016-12-12

**Authors:** MIRANDA R. BERTRAM, GABRIEL L. HAMER, BARRY K. HARTUP, KAREN F. SNOWDEN, MATTHEW C. MEDEIROS, SARAH A. HAMER

**Affiliations:** 1 Department of Veterinary Integrative Biosciences, Texas A&M University, 4458 TAMU, College Station, TX 77843, USA; 2 Department of Entomology, Texas A&M University, 2475 TAMU, College Station, TX 77843, USA; 3 International Crane Foundation, E-11376 Shady Lane Rd., Baraboo, WI 53913, USA; 4 Department of Surgical Sciences, University of Wisconsin, 2015 Linden Dr., Madison, WI 53706, USA; 5 Department of Veterinary Pathobiology, Texas A&M University, 4467 TAMU, College Station, TX 77843, USA

**Keywords:** Haemosporida, whooping crane, *Grus americana*, sandhill crane, *Grus canadensis*, molecular epizootiology, surrogate species

## Abstract

The population growth of endangered whooping cranes (*Grus americana*) is not consistent with species recovery goals, and the impact of parasite infection on whooping crane populations is largely unknown. Disease ecology and epidemiology research of endangered species is often hindered by limited ability to conduct invasive sampling on the target taxa. Accordingly, we hypothesized that sandhill cranes (*Grus canadensis*) would be a useful surrogate species to investigate the health impacts of Haemosporida infection in whooping cranes. Our goal was to compare the prevalence and diversity of Haemosporida infection between whooping cranes and sandhill cranes. We detected an overall infection prevalence of 83·6% (*n* = 61) in whooping cranes and 59·6% (*n* = 47) and 63·6 (*n* = 22) in two sympatric sandhill crane populations captured in Texas. Prevalence was significantly lower in allopatric sandhill cranes captured in New Mexico (12·1%, *n* = 33). *Haemoproteus antigonis* was the most abundant haemoparasite in cranes, present in 57·4% of whooping cranes and 39·2% of sandhill cranes; *Plasmodium* and *Leucocytozoon* were present at significantly lower levels. The high prevalence of Haemosporida in whooping cranes and sympatric sandhill cranes, with shared parasite lineages between the two species, supports sandhill cranes as a surrogate species for understanding health threats to endangered whooping cranes.

## INTRODUCTION

Whooping cranes (*Grus americana*) are an endangered species in North America, with the Aransas-Wood Buffalo population (AWBP) as the only self-sustaining wild population. This population nests in Wood Buffalo National Park, Alberta and Northwest Territories, Canada and winters among coastal marshes at the Aransas National Wildlife Refuge (ANWR) in Texas, USA. During winter 2015–2016, this population was estimated at 329 individuals (95% CI = 293–371; CV = 0·073) (Butler & Harrell, 2016), a substantial increase from a population low of 15 individuals. Population projections, however, indicate that whooping cranes may not achieve the down-listing criterion of a population size of 1000 individuals (Canadian Wildlife Service & US Fish and Wildlife Service, 2005) until the mid-2060s (Gil-Weir *et al.* 2012). Although parasitic infection may be one factor limiting population growth, species management efforts cannot specifically account for infections because very few studies have investigated parasites in the birds (but see Forrester *et al.* 1978; Bertram *et al.* 2015), likely reflecting the challenges of conducting infectious disease surveillance in an endangered species.

Acute Haemosporida infection has been shown to cause mortality in birds, and has been implicated in the decline and extinction of native Hawaiian bird species (Warner, 1968). Due to the short, transient nature of the acute stage of infection and limited mobility of infected hosts during this stage, most studies of Haemosporida infections in wild populations have investigated the chronic phase of infection, which is characterized by low parasitaemias and few or mild clinical signs. Although overt clinical signs are rare, chronic Haemosporida infections are increasingly associated with detrimental effects among infected individuals (Asghar *et al.* 2015). Prior studies of Haemosporida in cranes of North America are based on examination of blood smears and include descriptions of *Haemoproteus antigonis, Haemoproteus balearicae, Plasmodium-polare*-like and *Leucocytozoon grusi* in sandhill cranes (*Grus canadensis*) (Dusek *et al.* 2004), and *H. antigonis* in a small number of non-migratory whooping cranes in Florida (Forrester & Spalding, 2003). The majority of chronic Haemosporida infections reported in sandhill cranes have not been reported to cause disease; however, severe anaemia has been associated with acute *Haemoproteus balearicae* infection in two sandhill crane chicks (Dusek *et al.* 2004). Previous studies have reported *Haemoproteus* prevalence of 8–14% in mixed-age sandhill cranes from Florida and from Western North America (Forrester *et al.* 1974; Lee *et al.* 1985), and Dusek *et al.* (2004) reported a higher prevalence (36%) among sandhill crane chicks in Florida. We have identified *H. antigonis* infections in AWBP whooping cranes and sympatric sandhill cranes, which comprises a novel molecular clade formed by the parasite species (Bertram *et al.* unpublished data).

The paucity of health studies on the endangered whooping cranes is a barrier to their effective management and is due in part to the difficulty of obtaining robust sample sizes given permitting and ethical limitations for invasive or disruptive sampling that could negatively impact individuals. The surrogate species approach has been promoted in health studies of endangered taxa to provide otherwise unattainable data for use in assessing potential exposures and disease burden to guide species recovery plans. For example, in order to better understand threats to the endangered Attwater's prairie chicken, sympatric northern bobwhite were assessed for helminthic endoparasites and specific antibodies against infectious agent (Purvis *et al.* 1998), and a similar parasite community was revealed when compared with direct studies of the prairie chickens conducted earlier (Peterson *et al.* 1998). We suggest that the sandhill crane is a candidate surrogate species for the whooping crane because it is the closest North American relative to the whooping crane, it is abundant, and the Mid-continent population of sandhill cranes has an overlapping range with whooping cranes. We hypothesized that sympatric sandhill cranes will harbour a similar Haemosporida prevalence and diversity to whooping cranes, whereas allopatric sandhill cranes would be associated with a different Haemosporida infection profile. Our objective was to determine the prevalence of infection with Haemosporida (*Plasmodium, Haemoproteus* and *Leucocytozoon*) in AWBP whooping cranes and three different wintering populations of sandhill cranes with differing levels of sympatry to the whooping cranes.

## METHODS AND MATERIALS

### Sample collection

**Fig. 1.**
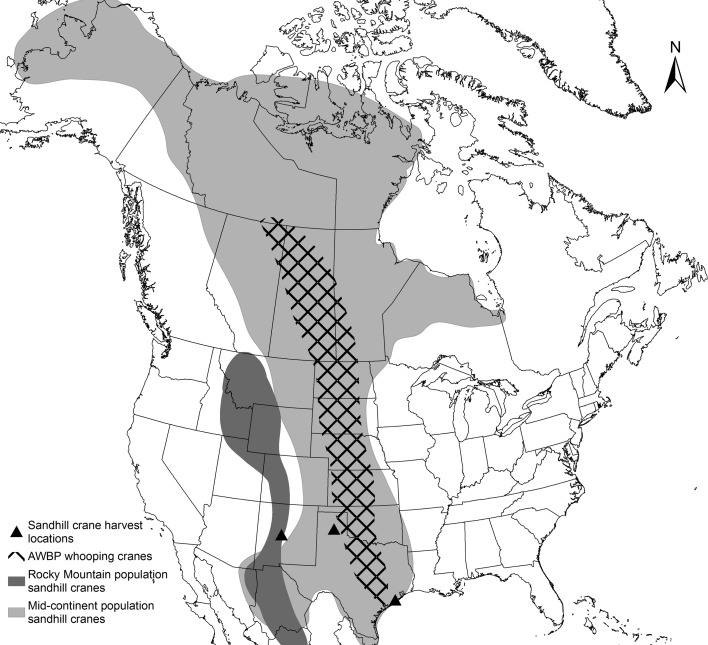
Ranges of the Mid-continent and Rocky Mountain populations of sandhill cranes in North America and AWBP whooping cranes. Ranges shown include breeding, winter and migration routes (Gil-Weir *et al.* 2012; Krapu *et al.* 2011). Locations where sandhill cranes included in this study were harvested are indicated.

Whooping crane blood samples were collected as part of an ongoing telemetry and health monitoring study of the AWBP whooping cranes between 2010 and 2014 (Pearse *et al.* 2015). On both the breeding grounds at Wood Buffalo National Park (Alberta and Northwest Territories, Canada) and the wintering grounds at ANWR (Texas Gulf Coast), birds were captured manually (pre-fledging juveniles) or using remote triggered leg snares that enclosed on the lower tarsus upon triggering. Birds were manually restrained under valid federal, state and provincial permits. Blood was drawn from the jugular vein, and an aliquot of whole blood preserved in Longmire's buffer [0·1 m Tris, 0·1 m EDTA (ethylenediaminetetraacetic acid), 0·01 m NaCl, 0·5% SDS (sodium dodecyl sulphate), pH 8·0] was used in this study. We collected blood samples from hunter-harvested sandhill cranes at necropsy between November 2012 and January 2014 through relationships with the Texas Parks and Wildlife Department, New Mexico Department of Game and Fish, and private hunting clubs and outfitters. Sandhill cranes from the following three populations were sampled: (1) Mid-continent population wintering on the Texas Gulf Coast (harvested in Jackson County, TX). (2) Mid-continent population wintering in the Texas panhandle (harvested in Armstrong and Carson Counties, TX). (3) Rocky Mountain population wintering in New Mexico (harvested in Socorro County, NM). Some birds harvested in New Mexico may also have been part of the Mid-continent population. The Mid-continent population is comprised of Lesser (*G. c. canadensis*) and Greater (*G. c. tabida*) subspecies, whereas the Rocky Mountain population is comprised of the Greater subspecies only (Krapu *et al.* 2011). The Rocky Mountain population serves as an out-group for comparison because their breeding, migration and wintering ranges do not overlap with whooping cranes (Fig. 1). All birds were either subjected to necropsy in the field within 6 h post-harvest, or frozen at −20 °C immediately post-harvest and subjected to necropsy in the laboratory at a later date. Each carcass was subjected to a full gross necropsy, at which time we collected whole blood or blood clot, which had pooled in the coelomic cavity. Blood samples were frozen at −20 °C until DNA extraction.

### Molecular detection of Haemosporida

#### DNA extraction

DNA was extracted from 100 *µ*L of whole blood or blood clot using the E.Z.N.A Tissue Extraction kit (Omega Biotek, Norcross, GA) following the manufacturer's instructions for tissue extraction with modifications, including an overnight lysis step at 55 °C and elution into 100 *µ*L of elution buffer.

#### Haemosporida screening

All DNA samples were screened for Haemosporida using three separate PCRs to target different genes and results were interpreted in parallel. A sample was considered positive for overall prevalence estimates, if it met the criteria for positivity on at least one assay. We used negative DNA extraction controls and no template controls in all PCR reactions and samples reported as positive were from clean reactions.

First, *Plasmodium* and *Haemoproteus* infections were detected using a nested PCR reaction targeting an approximately 500 bp region of the 3′ end of the mitochondrial cytochrome *b* (*cytb*) gene. The first PCR reaction used the primers 3932F (Fecchio *et al.* 2013) and DW4 (Perkins & Schall, 2002) at a concentration of 0·2 *µ*m in a 15 *µ*L reaction. Remaining reaction components consisted of 1× FailSafe PCR Premix E (Epicentre, Madison, WI), 0·15 *µ*L FailSafe enzyme, 0·1 *µ*g *µ*L^−1^ BSA and 1 *µ*L of sample template. The second PCR reaction used the primers 413F and 926R (Ricklefs *et al.* 2005) at a concentration of 0·2 *µ*m in a 15 *µ*L reaction. Remaining reaction components were identical to the first PCR, except 1 *µ*L of the product from the first PCR was used as the template. In both rounds of PCR, cycling parameters were as described by Fecchio *et al*. (2013). A sample collected from a northern cardinal (*Cardinalis cardinalis*) and known to be infected with *Plasmodium* was used as a positive control (Medeiros *et al.* 2013).

Second, we used a nested PCR reaction targeting an approximately 900 bp region of the mitochondrial cytochrome oxidase subunit I (*coI*) gene. The first PCR reaction used the primers *coI*/outerF and *coI*/outerR (Martinsen *et al.* 2008) at a concentration of 0·3 *µ*m in a 15 *µ*L reaction with remaining reaction components as outlined above. The second PCR used the primers *coI*/nestedF and *coI*/nestedR (Martinsen *et al.* 2008) at a concentration of 0·3 *µ*m in a 15 *µ*L reaction. Remaining reaction components were identical to the first PCR, except 1 *µ*L of the product from the first PCR, diluted 1:20, which was used as the template. In both rounds of PCR, cycling parameters were as described by Martinsen *et al*. (2008). The same positive control used for *cytb* PCR reactions was also used for *coI* PCR reactions.

Third, DNA samples were screened specifically for *Leucocytozoon* infections using a nested PCR reaction targeting an approximately 700 bp region of the mitochondrial *cytb* gene. The first PCR reaction used the primers DW2 and DW4 (Perkins & Schall, 2002) at a concentration of 0·4 *µ*m in a 15 *µ*L reaction. Remaining reaction components consisted of 1× FailSafe PCR Premix B (Epicentre, Madison, WI), 0·15 *µ*L FailSafe enzyme and 1 *µ*L of sample template. The second PCR used the primers LeucoF and LeucoR (Sehgal *et al.* 2006) at a concentration of 0·4 *µ*m in a 20 *µ*L reaction. Remaining reaction components consisted of 1× FailSafe PCR Premix B (Epicentre, Madison, WI), 0·2 *µ*L FailSafe enzyme, and 2 *µ*L of the product from the first PCR, diluted 1:20. In both rounds of PCR, cycling parameters were as described by Sehgal *et al*. (2006). DNA extracted from bird blood known to be positive for *Leucocytozoon* was obtained from Ravinder Sehgal at San Francisco State University, San Francisco, CA, and used as a positive control.

### Sequencing and phylogenetic analyses

Amplicons were purified using ExoSAP-IT (Affymetrix, Santa Clara, CA) according to manufacturer's instructions. Purified samples were submitted for bi-directional sequencing to Eton Bioscience Inc. (San Diego, CA). Chromatographs were examined manually for quality and to assess congruence of forward and reverse strands using Clustal W within Mega 6·0 (Tamura *et al.* 2013). Sequences with double nucleotide peaks were separated using phasing. In this process, sequences with double nucleotide peaks were manually separated into all possible combinations of nucleotides that could have resulted in both nucleotides at a single polymorphic site. The resulting sequences were retained for analysis to determine the likely strains represented in the sample, however sequences with double nucleotide peaks were excluded from phylogenetic analysis. Sequences generated in this study were compared with known Haemosporida sequences using the BLAST tool in GenBank and were aligned with the closest matches and additional publicly available avian Haemosporida species sequences representative of unique clades in previous studies (Perkins & Schall, 2002; Martinsen *et al.* 2008; Outlaw & Ricklefs, 2010). Samples were considered positive for prevalence estimates if a DNA sequence was obtained for which the identity matched most closely to a Haemosporida species in GenBank. Due to the sequence homology in the *cytb* gene across *Leucocytozoon, Plasmodium* and *Haemoproteus* species, the PCR assay used to detect *Leucocytozoon* has been shown to produce false positive results for *Leucocytozoon* due to the presence of *Plasmodium* or *Haemoproteus* (Szollosi *et al.* 2008). Accordingly, we considered a sample positive for *Leucocytozoon* infection only if a DNA sequence was obtained for which the identity matched most closely to a *Leucocytozoon* species in GenBank.

Phylogenetic relationships were analysed in Mega 6·0 using the maximum-likelihood method based on a general time reversible with gamma distribution (GTR+G) model of evolution using the bootstrap method with 1000 replicates (Hall, 2011). The model was selected based on fit estimated by the Akaike information criterion (AICc) and Bayesian information criterion (BIC). Each unique sequence produced during this project and utilized in the phylogenetic analysis was deposited in GenBank (Accession no. KX223847–KX223877); when sequences occurred more than once in the dataset, the accompanying notes in GenBank identify all samples that shared the same sequence.

### Statistical analysis

Statistical analysis was performed using SAS software version 9.4 (Cary, NC). Overall prevalence and confidence intervals were calculated accounting for clustering at the population level. The chi-squared (*χ*^2^) test and logistic regression were used to investigate the relationships between haemoparasite infection and population, age and sex. Fisher's exact test was used to compare *Leucocytozoon* prevalence among populations due to small numbers of positive samples.

## RESULTS

### Molecular screening

We collected blood samples from 163 individual cranes, including 61 AWBP whooping cranes, 47 sandhill cranes captured in the Texas Panhandle, 22 sandhill cranes captured along the Texas Gulf Coast and 33 sandhill cranes captured in New Mexico (Table 1). When results from all three Haemosporida assays were interpreted in parallel, we detected Haemosporida in 83·6% (95% CI 74·0, 93·2) of whooping cranes and 59·6% (95% CI 45·0, 74·1) and 63·6% (95% CI 41·8, 85·5) in the two sympatric sandhill crane populations. Infection prevalence was significantly lower in allopatric sandhill cranes (12·1%, 95% CI 0·4, 23·9). *Haemoproteus antigonis* was detected in a similar proportion of whooping crane samples (57·4%, 95% CI 44·6, 70·2) and samples from two sympatric sandhill crane populations (57·5%, 95% CI 42·8, 72,1; and 50%, 95% CI 27·3, 72·7, respectively). The prevalence of *H. antigonis* was significantly lower in allopatric sandhill cranes (6·1%, 95% CI 0·0, 14·7). Whooping crane samples had a higher prevalence of *Plasmodium* spp., than sandhill crane samples overall (29·5%, 95% CI 17·7, 41·3; and 6·9%, 95% CI 1·9, 11·9, respectively), and the prevalence of *Plasmodium* was not significantly different among the three sandhill crane populations. The prevalence of *Leucocytozoon* spp. was not significantly different between whooping cranes (4·9%, 95% CI 0·0, 10·5) and sandhill cranes overall (3·9%, 95% CI 0·1, 45·1) or among sandhill crane populations.

**Table 1.** Prevalence of Haemosporida in AWBP whooping cranes (WHCR) and three populations of sandhill cranes (SACR). Overall prevalences (%) are given for each species, and prevalences for age (juvenile or adult) and sex (male, female or unknown) are given for each population

Population	*N*	*Haemoproteus antigonis*	*Plasmodium*	*Leucocytozoon*	All Haemosporida
WHCR	61	57·4 (44·6, 70·2)	29·5 (17·7, 41·3)	4·9 (0·0, 10·5)	83·6 (74·0, 93·2)
Female	24	66·7	33·3	8·3	95·8
Male	26	46	64·6	0	76·9
Unknown	11	63·6	9·1	9·1	72·7
Juvenile	22	13·6	68·2	9·1	81·8
Adult	39	82·1	7·7	2·6	84·6
SACR	102	39·2 (29·6, 48·9)	6·9 (1·9, 11·9)	3·9 (0·1, 7·8)	45·1 (35·3, 54·9)
*Panhandle*	47	57·5 (42·8, 72·1)	4·3 (0·0, 10·3)	2·1 (0·0, 6·4)	59·6 (45·0, 74·1)
Female	6	33·3	0	16·7	33·3
Male	29	51·7	3·5	0	51·7
Unknown	12	83·3	8·3	0	91·7
Juvenile	32	65·6	4·3	3·1	68·8
Adult	15	40	0	0	40
*Gulf Coast*	22	50 (27·3, 72·7)	18·2 (0·7, 35·7)	9·1 (0·0, 22·1)	63·6 (41·8, 85·5)
Female	8	62·5	37·5	0	75
Male	14	42·9	7·1	14·3	57·1
Juvenile	5	80	20	0	80
Adult	17	41·2	17·7	11·8	58·8
*New Mexico*	33	6·1 (0·0, 14·7)	3 (0·0, 9·2)	3 (0·0, 9·2)	12·1 (0·4, 23·9)
Female	14	0	7·1	0	7·1
Male	19	10·5	0	5·3	15·8
Juvenile	3	0	0	0	0
Adult	30	6·7	3·3	3·3	13·3

The 95% confidence intervals are given in parentheses.

#### Haemoproteus antigonis

Among the four crane populations, we detected significantly fewer infections with *H. antigonis* in the New Mexico population of sandhill cranes than in the other three populations (*χ*^2^ = 26·99, df = 3, *P* < 0·0001) (Table 1). Age and sex were not significant predictors of infection; however, age and sex were retained in the regression model because previous studies in other avian species have found differences in infection prevalence between juveniles and adults (Bennett & Fallis, 1960; Mendes *et al.* 2005; Atkinson & Samuel, 2010; Hill *et al.* 2010), and sex was not distributed equally across populations in our data. When controlling for age and sex of the bird, the odds of infection were 22·9 (95% CI 4·8, 109·1), 27·4 (95% CI 5·1, 148·2) and 17·5 (95% CI 3·3, 92·6) times higher in AWBP whooping cranes, sandhill cranes captured in the Panhandle and along the Gulf Coast than in sandhill cranes captured in New Mexico, respectively. *Haemoproteus antigonis* infection was present in hatch-year birds, including three of 21 hatch-year whooping cranes captured at Wood Buffalo National Park, four of five hatch-year sandhill cranes captured along the Gulf Coast, and 21 of 32 hatch-year sandhill cranes captured in the Panhandle.

#### Plasmodium

Among the four crane populations, we detected significantly more infections with *Plasmodium* in the AWBP whooping cranes than in the three sandhill crane populations (*χ*^2^ = 17·87, df = 3, *P* = 0·001), and hatch-year birds were significantly more likely to be parasitaemic than adults (*χ*^2^ = 10·73, df = 1, *P* = 0·001). When controlling for population and sex of the bird, the odds of infection were 6·62 (95% CI 2·16, 20·32) times higher in hatch-year than in adult birds, and when controlling for age and sex the odds of infection were 34·4 (95% CI 4·0, 333), 1·35 (95% CI 0·3, 5·6) and 8·26 (95% CI 0·9, 71·4) times higher in the AWBP whooping cranes than in the Panhandle, Gulf Coast and New Mexico populations, respectively. *Plasmodium* infection was present in hatch-year birds, including 14 of 21 hatch-year whooping cranes captured at Wood Buffalo National Park, one of five hatch-year sandhill cranes captured along the Gulf Coast, and two of 32 hatch-year sandhill cranes captured in the Panhandle.

#### Leucocytozoon

We found an overall prevalence of 4·29% (95% CI 0·75, 7·84) of *Leucocytozoon* infection in cranes, and we did not detect a significant difference in prevalence among the four crane populations (Fisher's exact test, *P* = 0·601). *Leucocytozoon* infection was present in hatch-year birds, including two of 21 hatch-year whooping cranes captured at Wood Buffalo National Park, and one of 32 hatch-year sandhill cranes captured in the Panhandle.

### Comparison of PCR assays and phylogenetic analysis

The three primer sets produced different results for some samples with respect to parasitaemia (Table 2). Results agreed between the Haemosporida cyt *b* and coI assays for 117 (72%) samples, between the Haemosporida cyt *b* and *Leucocytozoon* cyt *b* assays for 116 (71%) samples, and between the Haemosporida coI and *Leucocytozoon* cyt *b* assays for 121 (74%) samples.

**Table 2.** Prevalence of Haemosporida detected by each PCR assay in 163 whooping crane and sandhill crane blood samples

	*Haemoproteus antigonis*		*Plasmodium*		*Leucocytozoon*	
Assay	Prevalence (%)	95% CI	Prevalence (%)	95% CI	Prevalence (%)	95% CI
*Plasmodium* cyt *b*	30·1	6·4, 53·7	1·2	0·0, 4·5	0·0	na
*Plasmodium* coI	21·5	1·1, 41·9	12·3	0·0, 31·8	0·0	na
*Leucocytozoon* cyt *b*	38	3·9, 72·2	10·43	0·0, 27·9	4·3	0·8, 7·8

#### Haemosporida cyt b

We obtained DNA sequences from the cyt *b* gene for 37 samples, which were included in the phylogenetic analysis. Upon manual examination of the chromatograph traces, an additional three samples had double nucleotide peaks, indicating mixed infections with two strains of the parasite. The three samples were from two whooping cranes and one sandhill crane from the Gulf Coast and had the same double nucleotide peak at the same base pair. Sequences for these samples were separated using phasing. We determined that these three samples were infected with both *H. antigonis* strains (KX223856 and KX223860).

**Fig. 2.**
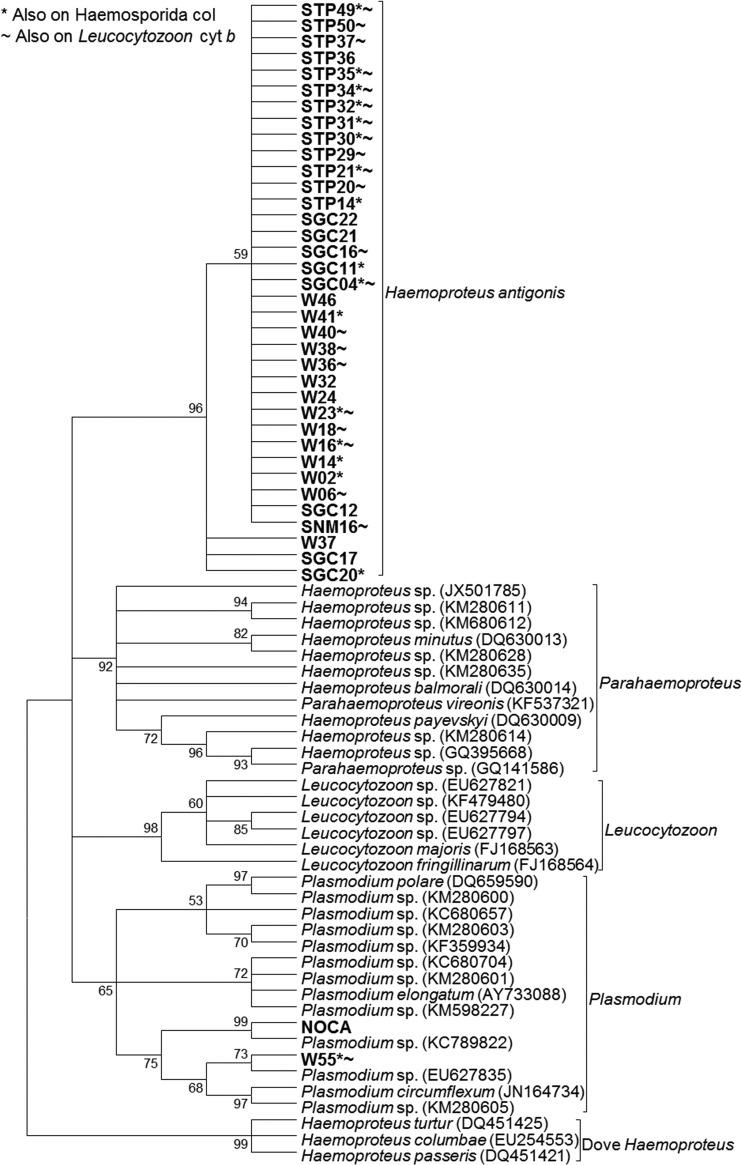
Cladogram using avian Haemosporida cyt *b* sequences (399 bp). The tree was created using the maximum-likelihood method with a GTR + G model of evolution. Bootstrap values are based on 1000 replicates, and nodes with <50% support are collapsed. Sequences in bold were generated in this study. NOCA – positive control sample from a northern cardinal. W – AWBP whooping crane, STP – sandhill crane harvested in the Texas, SGC – sandhill crane harvested on the Texas Gulf Coast, SNM – sandhill crane harvested in New Mexico.

Phylogenetic analysis revealed 33 sequences, identified as *H. antigonis*, were identical to each other (KX223856–KX223859) and formed a novel clade, along with an additional three sequences which each differed from the sequence of the majority clade at the same single base pair (KX223860–KX223861) (Fig. 2). *Haemoproteus antigonis* was detected in all four crane populations, and sequences from all four crane populations were identical to each other. Additionally, one sequence from a whooping crane sample aligned in a clade with a previously published *Plasmodium* sequence.

#### Haemosporida coI

We obtained sequences from the *coI* gene for 31 samples, which were included in the phylogenetic analysis. One additional sample had double nucleotide peaks, and phasing determined this sample was infected with two *H. antigonis* strains (KX223853 and KX223855).

**Fig. 3.**
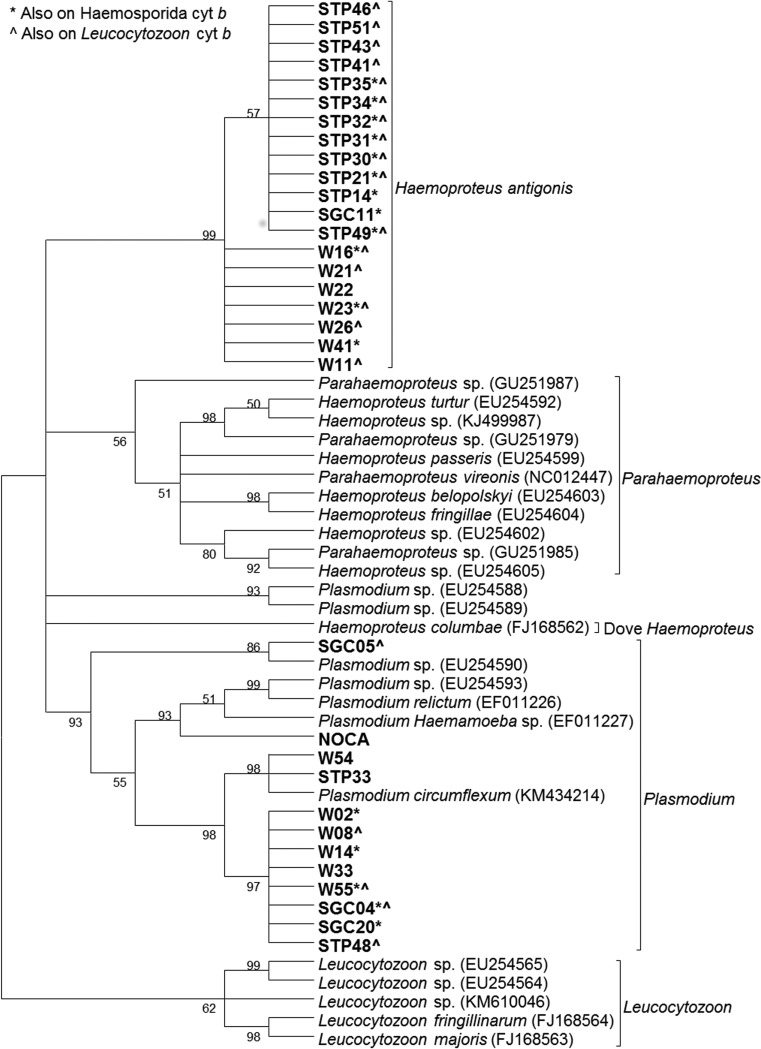
Cladogram using avian Haemosporida coI sequences (370 bp). The tree was created using the maximum-likelihood method with a GTR + G model of evolution. Bootstrap values are based on 1000 replicates, and nodes with <50% support are collapsed. Sequences in bold were generated in this study. NOCA – positive control sample from a northern cardinal. W – AWBP whooping crane, STP – sandhill crane harvested in the Texas, SGC – sandhill crane harvested on the Texas Gulf Coast, SNM – sandhill crane harvested in New Mexico.

Phylogenetic analysis revealed 13 identical *H. antigonis* sequences (KX223854–KX223855) that formed a novel clade along with seven additional sequences (KX223853), as was seen on the Haemosporida cyt *b* phylogenetic analysis (Fig. 3). One sequence aligned in a clade with a previously published *Plasmodium* sp. (Martinsen *et al.* 2008) sequence, two identical sequences aligned with a previously published *Plasmodium circumflexum* sequence, and an additional eight identical sequences formed a separate, closely related clade. Three sequences in this clade (W02, W14 and SGC20) aligned with *H. antigonis* in the Haemosporida cyt *b* analysis. AWBP whooping cranes and sandhill cranes captured in the Panhandle and along the Gulf Coast were represented in each of the unique clades, whereas sandhill cranes captured in New Mexico were not represented in the phylogenetic analysis for the coI assay.

#### Leucocytozoon cyt b

Although 74 of 163 samples produced a sequence from the *Leucocytozoon* PCR assay, a majority of the sequences (94%) revealed the presence of other Haemosporida species, which was not unexpected given the sequence homology across taxa (Szollosi *et al.* 2008). We obtained DNA sequences from the cyt *b* gene for 62 samples, which were included in the phylogenetic analysis. An additional 12 samples had double nucleotide peaks in the chromatographs. Ten samples, from whooping cranes and sandhill cranes captured along the Gulf Coast, had double nucleotide peaks at the same base pairs and were considered mixed infections with the two *H. antigonis* strains (KX223875 and KX223876). One sample had a mixed infection with two *Leucocytozoon* strains (KX223864 and KX223866), and one sample had a mixed infection with *Plasmodium* and *H. antigonis* (KX223867 and KX223873).

**Fig. 4.**
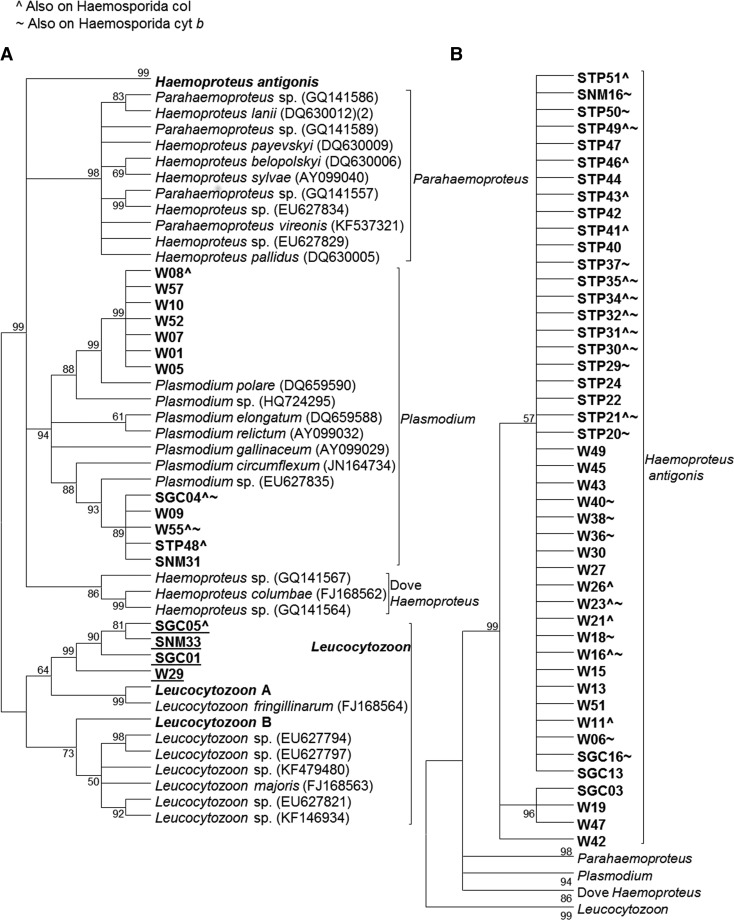
Cladogram using *Leucocytozoon* cyt *b* sequences (617 bp). A) Tree showing samples that do not fall in the novel crane Haemosporida clade. B) Tree showing samples that are included in the novel clade. The tree was created using the maximum-likelihood method with a GTR + G model of evolution. Bootstrap values are based on 1000 replicates, and nodes with <50% support are collapsed. Leucocytozoon A and B are positive control samples. Sequences in bold were generated in this study, and *Leucocytozoon* sequences are underlined. W – AWBP whooping crane, STP – sandhill crane harvested in the Texas, SGC – sandhill crane harvested on the Texas Gulf Coast, SNM – sandhill crane harvested in New Mexico.

Phylogenetic analysis revealed four sequences aligned with a clade containing previously published *Leucocytozoon* sequences; however, the crane sequences formed a distinct group within this clade (Fig. 4). All four crane populations were represented in this group. One sample in this group (SGC05) was also represented in the coI analysis, in which it grouped with *Plasmodium* sequences.

Of the 58 non-*Leucocytozoon* Haemosporida sequences obtained from this assay, 46 sequences, representing all four crane populations, were *H. antigonis* and formed a novel clade comprised two strains (KX223873–KX223875, KX223876–KX223877), consistent with results from our analyses of Haemosporida cyt *b* and coI. Seven were identical sequences (KX223867) and grouped with a previously published *Plasmodium polare* sequence. An additional five sequences (KX223869–KX223872) grouped with *P. circumflexum*. One sample in this clade (SGC04) also aligned with *Plasmodium* in the coI analysis, but aligned with *H. antigonis* in the Haemosporida cyt *b* analysis. The *P. polare* group consisted of whooping crane samples exclusively, whereas all four crane populations were represented in the other *Plasmodium* group.

Nine samples were represented in the *H. antigonis* clade in all three analyses, and an additional 21 samples were represented in the *H. antigonis* clade in two of the three analyses. One sample (W55) aligned with *Plasmodium* in all three analyses, and an additional two samples aligned with *Plasmodium* in two of the three analyses. All sequences represented in multiple analyses grouped with the same strain in each analysis, with the exception of the samples mentioned above, which aligned with different species in different analyses.

## DISCUSSION

A surrogate species is one that is expected to respond to environmental conditions in a manner similar to the species of interest, usually a species of conservation concern (Caro *et al*. 2005; Murphy *et al*. 2011).Surrogate species are often used to study endangered species when the endangered species is particularly susceptible to disturbance, individuals are difficult to locate, the population is too small to afford an adequate sample size, or when use of the endangered species would be impossible (e.g. experimental studies). We applied the surrogate species approach in a parasitological context to describe infections with Haemosporida in the genera *Haemoproteus, Plasmodium* and *Leucocytozoon* in endangered wild whooping cranes and three wintering populations of sandhill cranes. We found a similar prevalence and diversity of Haemosporida in whooping cranes and sympatric sandhill cranes, and markedly lower infection in allopatric sandhill cranes, supporting our hypothesis that sandhill cranes that co-occur with whooping cranes are appropriate surrogates.

We found a high prevalence (50–57%) of *H. antigonis* in AWBP whooping cranes and the two sympatric sandhill crane populations that winter in Texas. In contrast, the sandhill crane population wintering in New Mexico, which does not overlap with whooping cranes, had a significantly lower prevalence (6%) of *H. antigonis*. The lower prevalence in sandhill cranes harvested in New Mexico may reflect that the majority of sandhill cranes wintering in New Mexico are part of the Rocky Mountain population, which breeds in and around Southeastern Idaho, whereas the two Texas wintering sandhill crane populations are part of the Mid-continent population which breeds in Northern Canada.

Our observations include Haemosporida (*H. antigonis, Plasmodium* and *Leucocytozoon*) infection in juvenile whooping cranes sampled on their breeding grounds at Wood Buffalo National Park. This finding indicates that vector-borne transmission is occurring on the breeding grounds. We also noted infections in sympatric juvenile sandhill cranes harvested on the wintering grounds. The prepatent period for most Haemosporida is 11 days to three weeks (Valkiunas, 2005), and the sandhill cranes were harvested shortly after arrival to the wintering grounds, which suggests that transmission occurred prior to the arrival of the birds to the wintering grounds. These observations support previous studies, including one on sandhill cranes, which indicate birds usually acquire haemoparasite infections from biting Diptera on their breeding grounds (Lee *et al.* 1985).

The specific vector species are unknown for most avian Haemosporida, including those infecting cranes. Previous studies have suggested *Culicoides* midges (Bennett & Fallis, 1960) and Hippoboscid flies (Lee *et al.* 1985) are the primary vectors for *Parahaemoproteus* and *Haemoproteus*, respectively, and mosquitoes are the primary vectors for *Plasmodium* [reviewed by Valkiunas (2005)]. Interestingly, the most common haemosporidian parasite (*H. antigonis*) in this study infected all four crane populations, suggesting its vector has a broad geographic range. Despite the uncertainty of vector identity, whooping cranes and Mid-continent sandhill cranes are likely exposed to the same vector communities on their breeding grounds in Northern Canada, resulting in a similar prevalence and diversity of Haemosporida among whooping cranes and sympatric sandhill cranes and further supporting sandhill cranes as a surrogate species.

This is the first study to use molecular techniques to detect haemoparasite infection in North American cranes, and the prevalence reported here is higher than previous studies of sandhill cranes that relied on microscopy. Morphologic studies of Haemosporida are dependent on the quality of the blood smear and the search effort (Valkiunas *et al.* 2008) and may underestimate infection prevalence, especially in chronic infections with low parasitaemia (Garamszegi, 2010). Further, many Haemosporida are difficult to identify to species based on morphology, especially when infection intensity is low and not all stages of the parasite are represented in the sample. We used three different PCR assays in parallel to detect infection and mitigate differences in sensitivity and specificity among individual assays, which impact prevalence estimates determined by single assays (Garamszegi, 2010). Nonetheless, 46 of 97 positive samples were represented in at least two of the three phylogenetic analyses. Different primers may have differing affinities for different haemosporidian lineages, which can result in bias, especially when mixed infections are present. Additionally, when there are large differences in parasitaemia between co-infecting lineages, the consensus sequence may be biased towards the lineage with the higher concentration of DNA in the sample. In our study, the *coI* and *Leucocytozoon* cyt *b* primers resulted in a higher prevalence of *Plasmodium* than the Haemosporida cyt *b* primers. Further, our approach revealed mixed infections in seven samples with *H. antigonis* and *Plasmodium*, which may be missed by a single assay (Valkiunas *et al.* 2006). Our results underscore the importance of using multiple assays to increase probability of detection of avian Haemosporida and to help uncover mixed infections.

We detected a low prevalence of *Leucocytozoon* infection (2–9%) in the four crane populations, which constitutes the first report of *Leucytozoon* infection in whooping cranes and in sandhill cranes wintering in Texas and New Mexico. This infection prevalence is conservative because we required sequence confirmation of *Leucocytozoon* to consider a sample positive. Although we screened for coinfections as evidenced by double nucleotide peaks in the sequence chromatographs, we may have failed to detect coinfections, especially if another Haemosporida was present at higher levels than *Leucocytozoon*. A single species of *Leucocytozoon, L. grusi*, has been reported in sandhill cranes (Bennett *et al.* 1974). Previous studies, based on microscopy, have reported a prevalence of 8–18% in the non-migratory Florida sandhill cranes (Bennett *et al.* 1974; Forrester *et al.* 1975). In contrast, infection prevalence was 50% in one study examining Florida sandhill crane chicks only (Dusek *et al.* 2004). Forrester *et al.* (1974) did not detect *L. grusi* in any of 51 Greater (migratory) sandhill cranes wintering in Florida, and *Leucocytozoon* infection has not previously been reported in whooping cranes (Forrester & Spalding, 2003). The Florida sandhill cranes were likely infected during the summer, when Simuliid flies, the vector for *Leucocytozoon*, were abundant (Forrester & Spalding, 2003). Our observations of infections of *Leucocytozoon* in two juvenile whooping cranes sampled on the breeding grounds in Canada and one sandhill crane harvested in the Texas Panhandle provides further evidence that exposure is occurring during the summer on breeding grounds for both species.

We found many identical parasite sequences in samples from whooping and sandhill cranes, suggesting these parasites are shared between the two crane species. This is likely the result of generalist haematophagous arthropod vectors that feed opportunistically on either crane species as well as the parasite's propensity to infect closely related hosts (Medeiros *et al.* 2013; Ellis *et al.* 2015). This highlights the utility of sympatric sandhill cranes as a surrogate for the whooping crane. Given the establishment of baseline population-level infection prevalence data and validation of the surrogate species, future studies investigating the individual clinical impacts as well as population-level effects (e.g. altered survivorship or recruitment) of avian Haemosporida will aid in the management of both sandhill cranes and whooping cranes.

## References

[ref1] Asghar M., Hasselquist D., Hansson B., Zehtindjiev P., Westerdahl H. and Bensch S. (2015). Hidden costs of infection: chronic malaria accelerates telomere degradation and senescence in wild birds. Science 347, 436–438. 25613889 10.1126/science.1261121

[ref2] Atkinson C. T. and Samuel M. D. (2010). Avian malaria *Plasmodium relictum* in native Hawaiian forest birds: epizootiology and demographic impacts on ‘apapane *Himatione sanguinea*. Journal of Avian Biology 41, 357–366.

[ref3] Bennett G. F. and Fallis A. M. (1960). Blood parasites of birds in Algonquin Park, Canada, and a discussion of their transmission. Canadian Journal of Zoology 38, 261–273.

[ref4] Bennett G. F., Khan R. A. and Campbell A. G. (1974). *Leucocytozoon grusi* sp. n. (Sporozoa: Leucocytozoidae) from a sandhill crane, *Grus canadensis* (L.). Journal of Parasitology 60, 359–363. 4206882

[ref6] Bertram M. R., Hamer G. L., Snowden K., Hartup B. K. and Hamer S. A. (2015). Coccidian parasites and conservation implications for the endangered whooping crane (*Grus americana*). PLoS ONE 10, e0127679. 26061631 10.1371/journal.pone.0127679 PMC4464527

[ref7] Butler M. J. and Harrell W. (2016). Whooping crane survey results: Winter 2015–2016. Vol. 2016 USFWS.

[ref8] Canadian Wildlife Service and US Fish and Wildlife Service (2005). International Recovery Plan for the Whooping Crane. Recovery of Nationally Endangered Wildlife (RENEW), and U.S. Fish and Wildlife Service, Ottawa, Albuquerque, NM pp. 162.

[ref5a] Caro T., Eadie J. and Sih A. (2005). Use of substitute species in conservation biology. Conservation Biology 19, 1821–1826.

[ref9] Dusek R. J., Spalding M. G., Forrester D. J. and Greiner E. C. (2004). *Haemoproteus balearicae* and other blood parasites of free-ranging Florida sandhill crane chicks. Journal of Wildlife Diseases 40, 682–687. 15650085 10.7589/0090-3558-40.4.682

[ref10] Ellis V. A., Collins M. D., Medeiros M. C., Sari E. H., Coffey E. D., Dickerson R. C., Lugarini C., Stratford J. A., Henry D. R., Merrill L., Matthews A. E., Hanson A. A., Roberts J. R., Joyce M., Kunkel M. R. and Ricklefs R. E. (2015). Local host specialization, host-switching, and dispersal shape the regional distributions of avian haemosporidian parasites. Proceedings of the National Academy of Sciences of the United States of America 112, 11294–11299. 26305975 10.1073/pnas.1515309112 PMC4568705

[ref11] Fecchio A., Lima M. R., Svensson-Coelho M., Marini M. A. and Ricklefs R. E. (2013). Structure and organization of an avian haemosporidian assemblage in a Neotropical savanna in Brazil. Parasitology 140, 181–192. 22939119 10.1017/S0031182012001412

[ref12] Forrester D. J. and Spalding M. G. (2003). Parasites and Diseases of Wild Birds in Florida, University Press of Florida, Gainesville, FL.

[ref13] Forrester D. J., Bush A. O., Williams L. E. and Weiner D. J. (1974). Parasites of greater sandhill cranes (*Grus canadensis tabida*) on their wintering grounds in Florida. Proceedings of the Helminthological Society of Washington 41, 55–59.

[ref14] Forrester D. J., Bush A. O. and Williams L. E. (1975). Parasites of Florida sandhill cranes (*Grus canadensis pratensis*). Journal of Parasitology 61, 547–548.

[ref15] Forrester D. J., Carpenter J. W. and Blankinship D. R. (1978). Coccidia of whooping cranes. Journal of Wildlife Diseases 14, 24–27. 633514 10.7589/0090-3558-14.1.24

[ref16] Garamszegi L. Z. (2010). The sensitivity of microscopy and PCR-based detection methods affecting estimates of prevalence of blood parasites in birds. Journal of Parasitology 96, 1197–1203. 21158636 10.1645/GE-2531.1

[ref17] Gil-Weir K. C., Grant W. E., Slack R. D., Wang H.-H. and Fujiwara M. (2012). Demography and population trends of Whooping Cranes. Journal of Field Ornithology 83, 1–10.

[ref18] Hall B. G. (2011). Phylogenetic Trees Made Easy: A How to Manual, 4th Edn. Sinauer Associates, Inc., Sunderland, MA.

[ref19] Hill A. G., Howe L., Gartrell B. D. and Alley M. R. (2010). Prevalence of *Leucocytozoon* spp, in the endangered yellow-eyed penguin *Megadyptes antipodes*. Parasitology 137, 1477–1485. 20557665 10.1017/S0031182009991910

[ref20] Krapu G. L., Brandt D. A., Jones K. L. and Johnson D. H. (2011). Geographic distribution of the mid-continent population of sandhill cranes and related management applications. Wildlife Monographs 175, 1–38.

[ref21] Lee S. D., Pence D. B. and Gaines G. D. (1985). *Haemoproteus antigonis* from the sandhill crane in western North America. Proceedings of the Helminthological Society of Washington 52, 311–312.

[ref22] Martinsen E. S., Perkins S. L. and Schall J. J. (2008). A three-genome phylogeny of malaria parasites (*Plasmodium* and closely related genera): evolution of life-history traits and host switches. Molecular Phylogenetics and Evolution 47, 261–273. 18248741 10.1016/j.ympev.2007.11.012

[ref23] Medeiros M. C., Hamer G. L. and Ricklefs R. E. (2013). Host compatibility rather than vector-host-encounter rate determines the host range of avian Plasmodium parasites. Proceedings of the Royal Society B: Biological Sciences 280, 2012–2947. 10.1098/rspb.2012.2947 PMC3652452 23595266

[ref24] Mendes L., Piersma T., Lecoq M., Spaans B. and Ricklefs R. E. (2005). Disease-limited distributions? Contrasts in the prevalence of avian malaria in shorebird species using marine and freshwater habitats. Oikos 109, 396–404.

[ref24a] Murphy D. D., Weiland P. S. and Cummins K. W. (2011). A critical assessment of the use of surrogate species in conservation planning in the Sacreamento-San Joaquin Delta, California (USA). Conservation Biology 25, 873–878. 21790783 10.1111/j.1523-1739.2011.01711.x

[ref25] Outlaw D. C. and Ricklefs R. E. (2010). Comparative gene evolution in haemosporidian (*Apicomplexa*) parasites of birds and mammals. Molecular Biology and Evolution 27, 537–542. 19933837 10.1093/molbev/msp283

[ref26] Pearse A. T., Brandt D. A., Harrell W. C., Metzger K. L., Baasch D. M. and Hefley T. J. (2015). Whooping crane stopover site use intensity within the Great Plains. http://pubs.er.usgs.gov/publication/ofr20151166 (September 25). 10·3133/ofr20151166.

[ref27] Perkins S. L. and Schall J. J. (2002). A molecular phylogeny of malarial parasites recovered from cytochrome *b* gene sequences. Journal of Parasitology 88, 972–978. 12435139 10.1645/0022-3395(2002)088[0972:AMPOMP]2.0.CO;2

[ref28] Peterson M. J., Purvis J. R., Lichtenfels J. R., Craig T. M., Dronen N. O. J. and Silvy N. J. (1998). Serologic and parasitologic survey of the endangered Attwater's prairie chicken. Journal of Wildlife Diseases 34, 137–144. 9476235 10.7589/0090-3558-34.1.137

[ref29] Purvis J. R., Peterson M. J., Dronen N. O., Lichtenfels J. R. and Silvy N. J. (1998). Northern bobwhites as disease indicators for the endangered Attwater's prairie chicken. Journal of Wildlife Diseases 34, 348–354. 9577783 10.7589/0090-3558-34.2.348

[ref30] Ricklefs R. E., Swanson B. L., Fallon S. M., Martinez-Abrain A., Scheuerlein A., Gray J. and Latta S. C. (2005). Community relationships of avian malaria parasites in southern Missouri. Ecological Monographs 75, 543–559.

[ref31] Sehgal R. N., Hull A. C., Anderson N. L., Valkiunas G., Markovets M. J., Kawamura S. and Tell L. A. (2006). Evidence for cryptic speciation of *Leucocytozoon* spp. (Haemosporida, Leucocytozoidae) in diurnal raptors. Journal of Parasitology 92, 375–379. 16729697 10.1645/GE-656R.1

[ref32] Szollosi E., Hellgren O. and Hasselquist D. (2008). A cautionary note on the use of nested PCR for parasite screening – an example from avian blood parasites. Journal of Parasitology 94, 562–564. 18564767 10.1645/GE-1286.1

[ref33] Tamura K., Stecher G., Peterson D., Filipski A. and Kumar S. (2013). MEGA6: molecular Evolutionary Genetics Analysis version 6.0. Molecular Biology and Evolution 30, 2725–2729. 24132122 10.1093/molbev/mst197 PMC3840312

[ref34] Valkiunas G. (2005). Avian Malaria Parasites and Other Haemosporidia, CRC Press, Boca Raton, FL.

[ref35] Valkiunas G., Bensch S., Iezhova T. A., Krizanauskiene A., Hellgren O. and Bolshakov C. V. (2006). Nested cytochrome B polymerase chain reaction diagnostics underestimate mixed infections of avian blood haemosporidian parasites: microscopy is still essential. Journal of Parasitology 92, 418–422. 16729711 10.1645/GE-3547RN.1

[ref36] Valkiunas G., Lezhova T. A., Krizanauskiene A., Palinauskas V., Sehgal R. N. and Bensch S. (2008). A comparative analysis of microscopy and PCR-based detection methods for blood parasites. Journal of Parasitology 94, 1395–1401. 18576856 10.1645/GE-1570.1

[ref37] Warner R. E. (1968). Role of introduced diseases in extinction of endemic Hawaiian avifauna. Condor 70, 101–120.

